# Antioxidant effects of continuous intake of electrolyzed hydrogen water in healthy adults

**DOI:** 10.1016/j.heliyon.2022.e11853

**Published:** 2022-11-25

**Authors:** Kei Mizuno, Kyosuke Watanabe, Emi Yamano, Kyoko Ebisu, Kanako Tajima, Junzo Nojima, Yusuke Ohsaki, Shigeru Kabayama, Yasuyoshi Watanabe

**Affiliations:** a Laboratory for Pathophysiological and Health Science, RIKEN Center for Biosystems Dynamics Research, 6-7-3 Minatojima-minamimachi, Chuo-ku, Kobe City, Hyogo 650-0047, Japan; b RIKEN Compass to Healthy Life Research Complex Program, 6-7-1 Minatojima-minamimachi, Chuo-ku, Kobe City, Hyogo 650-0047, Japan; c Osaka City University Center for Health Science Innovation, 3-1 Ofuka-cho, Kita-ku, Osaka City, Osaka 530-0011, Japan; d Department of Laboratory Science, Faculty of Health Science, Yamaguchi University Graduate School of Medicine, 1-1-1 Minami-kogushi, Ube City, Yamaguchi 755-8505, Japan; e Laboratory of Nutrition, Graduate School of Agricultural Science, Tohoku University, 468-1 Aramaki Aza Aoba, Aoba-ku, Sendai City, Miyagi 980-8572, Japan; f Trim Medical Institute Co. Ltd., 22F HERBIS ENT Office Tower 2-2-22 Umeda, Osaka City, Osaka 530-0001, Japan

**Keywords:** Electrolyzed hydrogen water, Antioxidant oxidative stress, Inflammation, LDL cholesterol, Renal function

## Abstract

Chronic oxidative stress induces deterioration of health and a risk for the onset of various diseases. Previous clinical studies revealed that electrolyzed hydrogen water (EHW) is effective to reduce oxidative stress during hemodialysis in patients with chronic dialysis. In the present observational study, we investigated the antioxidant effects of a daily continuous intake of EHW in healthy adults. The concentrations of serum reactive oxygen metabolites-derived compounds (d-ROMs) and blood urea nitrogen in healthy volunteers (n = 64) who had a habit of intake over 500 mL/day of EHW at least 5 days a week for longer than 6 months were lower than those of age- and sex-matched controls (n = 470) without the habit of EHW intake. Oxidation stress index which the ratio between concentrations in d-ROMs and biological antioxidant potential was correlated with the serum concentration of high-sensitivity C-reactive protein or low-density lipoprotein cholesterol in the EHW group. These results suggest that the continuous intake of EHW induces antioxidant effects and may contribute to alleviate the risk of various oxidative stress-related dysfunctions and diseases in healthy adults.

## 1 Introduction

Oxidative stress induced by generation of excess reactive oxygen species (ROS) during energy production for work or over work could result in the pathophysiology of a variety of diseases (cardiovascular diseases, chronic obstructive pulmonary disease, chronic kidney disease, neurodegenerative diseases, and cancer) [1]. To prevent the onset of these diseases, daily or scheduled intake of the antioxidant nutrients derived from water, food, and supplements is important.

Electrolytic hydrogen water (EHW) contains abundant molecular hydrogen and very small amounts of platinum nanoparticles with alkaline properties and has a scavenging activity of antioxidants toward ROS [2]. In our recent study, EHW intake of rats with chronic stress induces decrease in the serum concentration of reactive oxygen metabolites-derived compounds (d-ROMs) and oxidative stress index (OSI) as markers of oxidative stress [3]. The OSI is calculated using the following formula: OSI = C [d-ROMs/biological antioxidant potential (BAP)] where C denotes a coefficient for standardization to set the mean OSI in healthy individuals at 1.0 (C = 8.85) [4]. We investigated these antioxidant effects of a daily continuous intake of EHW in the present study. In our clinical studies, EHW administration diminished hemodialyis-enhanced oxidative stress during 1- and 6-month treatments in patients with end-stage renal disease [5, 6]. Renal function including the dynamics of blood urea nitrogen (BUN) is related to the oxidative stress [7]. Other studies revealed that an effectiveness of EHW on insulin resistance of diabetes [8, 9] and ethanol-induced cytotoxicity in hepatocytes [10]. However, the antioxidant effects of EHW intake in healthy individuals are still unclear. Accumulation of stress and fatigue in daily life of healthy volunteers induces increase in concentration of d-ROMs and decrease in BAP [11]. These facts suggest that the daily intake of EHW could inhibit the oxidative stress caused by a variety of workloads and decrease the future risk of oxidative stress-related diseases in healthy individuals. The aim of the present study was to evaluate the state of oxidative stress and the potential of anti-oxidative stress in healthy adults with the habit of daily intake of EHW.

## 2 Materials and methods

The EHW is a one of functional water which is generated from medical device for home-use named Alkaline Ionized Water Apparatus. The purpose of use and beneficial effects were reported in the Ministry of Health, Labour and Welfare Notification No. 112 as”generation of alkaline electrolytic drinking water for improving gastrointestinal symptoms”. According to the Statistical Survey on Trends in Pharmaceutical Production (from the Health Policy Bureau of the Ministry of Health and Welfare in Japan), there are reports that in recent years the total quantity of shipments in the entire industry reached about 200,000 annually. EHW contains abundant molecular hydrogen from 100 to 1,300 ppb and very small amounts of platinum nanoparticles with alkaline properties from pH 9.0 to 10.0 due to electrolysis of tap water. The level of pH and concentration of molecular hydrogen (H_2_) are adjustable by selecting buttons for the level of electrolysis and water flow rate. Intake method is to try to drink the freshest possible EHW generated from the apparatus. The pH is stable, on the other hand, the concentration of molecular hydrogen gradually decreases, to cite few cases, from 323 ppb to 300 ppb (7% down), from 894 ppb to 540 ppb (40% down) in stainless steel water bottle with filled it up after 12 h.

Healthy volunteers between 30 and 59 years of age were participated in the present observational study. Common exclusion criteria in control (CTL) and EHW groups comprised: history of chronic illness; chronic medication or use of supplemental vitamins; pregnancy; or history of smoking. Sixty-four participants in EHW group had the habit of intake over 500 mL/day of EHW (manufacturing equipment: Trim Ion Grace/Hyper/Neo, Nihon Trim Co., Ltd.; Osaka, Japan) at least 5 days a week for longer than 6 months. Four hundred and seventy participants in CTL group had the non-habit forming of EHW nor hydrogen-enriched (bubbled) water intake. Each participant was told to finish diet 4 h prior to the start of the collection of blood sample. The study protocol was approved by the Ethics Committee of RIKEN {Kobe2 2019-06 (2)} and all participants provided written informed consent for participation in the study. The study was undertaken in compliance with national legislation and the Code of Ethical Principles for Medical Research Involving Human Subjects of the World Medical Association (Declaration of Helsinki).

Blood samples were collected from the brachial vein and collected into EDTA blood collection tubes. Blood samples were analyzed on automatic biochemical analyzer for low-density lipoprotein (LDL) cholesterol, high-density lipoprotein (HDL) cholesterol, triglyceride, BUN, uric acid, glucose, hemoglobin A_1c_, aspartate aminotransferase, alanine aminotransferase, gamma-glutamyl transpeptidase, total protein, hemoglobin, creatinine kinase, and high-sensitivity C-reactive protein (hs-CRP). Blood samples for serum analyses were centrifuged at 1,470 × g at 4 °C for 5 min. The oxidative activity of serum samples was determined using a test for d-ROMs (Diacron International, Grosseto, Italy). The antioxidative activity was determined by measuring the BAP (Diacron International) using a JCABM1650 automated analyzer (JEOL, Tokyo, Japan). All supernatants were stored at −80 °C until analyzed. The period for the measurement of the testing blood for d-ROMs and BAP was less than one month after collecting the blood sample. The OSI was calculated using the following formula: OSI = C (d-ROMs/BAP) where C denotes a coefficient for standardization to set the mean OSI in healthy individuals at 1.0 (C = 8.85) [4].

Each measured value was represented as the mean ± standard deviation. Data analyses were performed by Student's *t*-test. Pearson's correlation analyses were also performed. Analysis for categorical data was conducted by chi-squared test. All *p*-values < 0.05 were considered statistically significant. Statistical analyses were performed using IBM SPSS Statistical Package version 27.0 (IBM, Armonk, NY, USA).

## 3 Results

**Figure 1:**
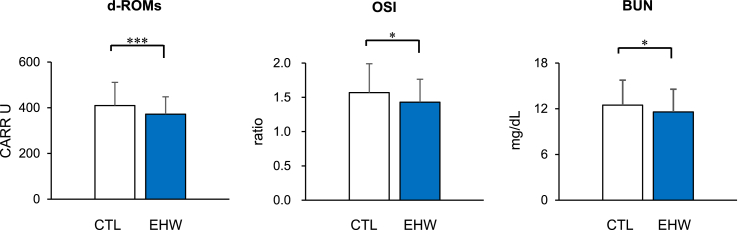
Effect of the continuous intake of electrolyzed hydrogen water (EHW) in daily life on oxidative stress and renal function. Levels of reactive oxygen metabolites-derived compounds (d-ROMs), oxidative stress index (OSI) and blood urea nitrogen (BUN) of the EHW group were reduced in comparison with the control (CTL) group. Error bars represent standard deviations. ∗∗∗*P* < 0.001, ∗*P* < 0.05, significant difference between the corresponding values.

**Figure 2:**
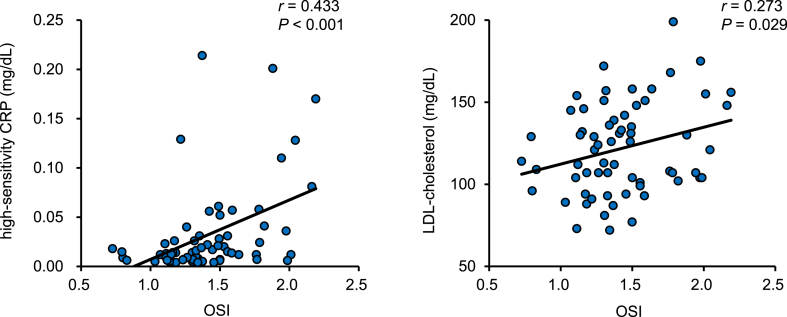
Correlations between oxidative stress and inflammation or cholesterol. Positive correlations between oxidative stress index (OSI) level and high-sensitivity C-reactive protein (CRP) level or low-density lipoprotein cholesterol (LDL-cholesterol) level in the group of electrolyzed hydrogen water were observed. Pearson's correlation coefficients (*r*) and *P*-values are shown.

Table 1 shows the demographic characteristics of the study participants and results of blood sample analyses. Sex, age, and body mass index between CTL and EHW groups were similar. Although the values of some biochemical parameters between the CTL and EHW groups were not significantly different (Table 1), the values of d-ROMs, OSI, and BUN in the EHW group were significantly lower than those in the CTL group (Figure 1). In order to clarify the associations between the extent of decrease in oxidative stress by continuous EHW intake and other blood parameters, we performed the correlation analyses in the EHW group. The value of OSI in the EHW group was positively correlated with that of hs-CRP or LDL cholesterol (Figure 2).

**Table 1:** Non-significant differences in basic and blood parameters between control (CTL) and electrolyzed hydrogen water (EHW) groups.

	CTL	EHW	*P*-value
Sex female (%)	390 (82.9)	52 (81.3)	0.731
Age (years)	43.4 ± 8.1	43.5 ± 7.6	0.949
BMI (kg/m^2^)	21.3 ± 3.1	21.6 ± 3.3	0.395
BAP (μM)	2331 ± 181	2329 ± 213	0.946
hs-CRP (mg/dL)	0.067 ± 0.027	0.065 ± 0.027	0.963
LDL (mg/dL)	120.4 ± 29.1	122.0 ± 27.5	0.690
HDL (mg/dL)	71.8 ± 17.3	70.2 ± 14.2	0.460
Triglyceride (mg/dL)	77.8 ± 50.2	69.3 ± 28.5	0.121
Uric acid (mg/dL)	4.41 ± 1.15	4.65 ± 1.15	0.188
Glucose (mg/dL)	84.5 ± 6.3	83.3 ± 6.0	0.135
HbA_1c_ (%)	5.30 ± 0.26	5.34 ± 0.23	0.337
AST (IU/L)	20.0 ± 6.8	20.0 ± 9.9	0.945
ALT (IU/L)	17.3 ± 13.0	17.0 ± 17.9	0.832
γ-GTP (IU/L)	22.9 ± 22.9	21.7 ± 19.3	0.694
Total protein (g/dL)	7.22 ± 0.39	7.24 ± 0.44	0.732
Hemoglobin (g/dL)	13.4 ± 1.3	13.6 ± 1.2	0.117
CK (IU/L)	100.7 ± 82.0	93.5 ± 44.1	0.493

BMI, body mass index; BAP, biological antioxidant potential; hs-CRP, high-sensitivity C-reactive protein; LDL, low-density lipoprotein cholesterol; HDL, high-density lipoprotein cholesterol; HbA_1c_, hemoglobin A_1c_; AST, aspartate aminotransferase; ALT, alanine aminotransferase; γ-GTP, gamma-glutamyl transpeptidase; CK, creatinine kinase. Values are shown as the mean ± standard deviation or number (%).

In supplemental analyses of the CTL group, the value of OSI in the CTL group was positively correlated with that of hs-CRP (*r* = 0.226, *p* < 0.001) and not with that of LDL cholesterol (*r* = 0.052, *p* = 0.261).

## 4 Discussion

Antioxidant effects of EHW on some diseases including renal disease and diabetes were demonstrated [5, 6, 9]. The findings of the present study are that the continuous intake of EHW is effective to reduce oxidative stress even in healthy individuals, not only the patients with oxidative stress-related diseases. We recently reported that elevated oxidative stress is attenuated by EHW administration of rats with chronic stress or fatigue [3]. In our human study, an increase in concentration of d-ROMs in healthy adults with accumulation of stress and fatigue is observed [11]. Specifically, the serum concentration of d-ROMs in healthy volunteers was increased after a computer-based mental arithmetic stress task for 3 h as an acute fatigue load. In addition, the serum level of d-ROMs in computer programmers was also increased after the overwork from 8:00 AM to overnight for two weeks as a sub-acute fatigue load. In the effect of EHW treatment to fatigue, a decrease in severity of fatigue in chronic dialysis patients by treatment of dialysis solution employing EHW for 8 weeks is confirmed [12]. These results suggest the preventing effects of continuous intake of EHW on stress and fatigue due to daily workload in healthy individuals.

Fukui and colleague [13] previously reported that the level of d-ROMs in 442 healthy volunteers was positively correlated with visceral adipose tissue evaluated by a computed tomography scan. A cross-sectional study reported that healthy individuals with excess visceral adipose tissue have several features of metabolic syndrome such as insulin resistance, high blood pressure, and inflammation [14]. In addition, several longitudinal studies demonstrated that visceral adipose tissue is a risk factor for metabolic syndrome [15, 16, 17]. These findings suggest that attenuation of oxidative stress due to continuous EHW intake in healthy individuals is effective to decrease in the risk of incidence of oxidative stress-related diseases such as metabolic syndrome.

Since oxidative stress is a trigger of various diseases (cardiovascular diseases, chronic obstructive pulmonary disease, chronic kidney disease, neurodegenerative diseases, and cancer) [1], daily intake of EHW may be effective to prevent the onset of these diseases. In the present study, we found the correlation between oxidative stress (OSI) and inflammation (hs-CRP). This correlation is also observed in patients with metabolic syndrome [18]. In addition, OSI was positively associated with the concentration of LDL cholesterol in the present study. Oxidation of LDL cholesterol is crucial in the development of atherosclerosis, and low level of LDL cholesterol reduce the risk for cardiovascular disease [19]. Excessive production of ROS and inflammation are often observed during the stress response and have been implicated in the progression of chronic diseases [20]. We confirmed that not only oxidative stress but also inflammation is inhibited by EHW intake in rats with chronic stress and fatigue [3]. These findings suggest that continuous intake of EHW in healthy adults is defensively affected to the pathways of ROS-induced inflammation in the progression of several pathologies.

The extent of oxidative stress relates to the renal function including the dynamics of BUN [7]. In chronic kidney disease, oxidative stress plays a pivotal role in pathologies [21]. Our previous and recent studies demonstrated the advanced antioxidant effects and reduced symptom severities in chronic dialysis patients by treatment of dialysis solution employing EHW [5, 6, 12]. These indicate that the present finding of decrease in BUN concentration by continuous intake of EHW may contribute to keep the better state of renal function.

The present study has several limitations. The information of regular diet of participants and the severities of chronic stress and fatigue were not investigated in the present study. Our review paper reported that autonomic nerve function evaluated by heart rate variability is suitable as a biological marker for evaluation of chronic stress and fatigue [22]. In addition, we did not directly examine using an intervention study to evaluate antioxidant effects of continuous intake of EHW. Therefore, using a randomized placebo-controlled trial, we are now conducting to examine the intervention effects of continuous EHW intake for several months while checking the regular diet of participants and measuring autonomic nerve function. And also in the prospective cohort study with daily intake of EHW for more than ten years in a few thousand people we would like to demonstrate less incidence of oxidative stress-related diseases by daily intake of EHW with the precise evaluation of well-being scores subjectively and objectively.

In conclusion, higher antioxidant potential was observed in healthy adult volunteers who had a habit of intake over 500 mL/day of EHW at least 5 days a week for longer than 6 months, suggesting the beneficial effects of EHW continuous intake for prevention from onset of a variety of oxidative stress-related diseases.

## Declarations

### Author contribution statement

Kei Mizuno, Kyosuke Watanabe, Yasuyoshi Watanabe: Conceived and designed the experiments; Performed the experiments; Analyzed and interpreted the data; Wrote the paper.

Emi Yamano, Kanako Tajima: Performed the experiments.

Kyoko Ebisu, Junzo Nojima: Performed the experiments; Analyzed and interpreted the data.

Yusuke Ohsaki: Conceived and designed the experiments; Performed the experiments.

Shigeru Kabayama: Conceived and designed the experiments; Performed the experiments; Wrote the paper.

### Funding statement

This work was supported by Nihon Trim Co., Ltd (2022-0250) and RIKEN Compass to Healthy Life Research Complex Program (RC-01) of the Japan Science and Technology Agency (JST).

### Data availability statement

Data will be made available on request.

### Declaration of interest's statement

The authors declare the following conflict of interests: Y.W. received the fund for the present study from Nihon Trim Co., Ltd. S.K., PhD, is the employee of Trim Medical Institute Co. Ltd. and the head of research group. The other authors declare that there are no conflicts of interest. The funding sponsors had no role in the design of the study; in the collection, analyses, or interpretation of data; in the writing of the manuscript, and in the decision to publish the results.

### Additional information

No additional information is available for this paper.
